# Evaluation of the Structure–Function Relationship of SGNH Lipase from *Streptomyces rimosus* by Site-Directed Mutagenesis and Computational Approach †

**DOI:** 10.3390/ijms25010595

**Published:** 2024-01-02

**Authors:** Želimira Filić, Ana Bielen, Ela Šarić, Mirsada Ćehić, Ivo Crnolatac, Sanja Tomić, Dušica Vujaklija, Marija Abramić

**Affiliations:** 1 Division of Physical Chemistry, Institute Ruđer Bošković, 10000 Zagreb, Croatia; zelimira.filic@irb.hr (Ž.F.); ela.saric@irb.hr (E.Š.); mirsada@vss.hr (M.Ć.); 2 Faculty of Food Technology and Biotechnology, University of Zagreb, 10000 Zagreb, Croatia; ana.bielen@pbf.unizg.hr; 3 Division of Organic Chemistry and Biochemistry, Institute Ruđer Bošković, 10000 Zagreb, Croatia; ivo.crnolatac@irb.hr (I.C.); marija.abramic@irb.hr (M.A.)

**Keywords:** SGNH/GDSL-hydrolase, site-directed mutagenesis, catalytic residues, molecular docking, molecular dynamics, protein–substrate interactions, substrate binding site, catalytic efficiency, *Streptomyces rimosus*

## Abstract

*Streptomyces rimosus* extracellular lipase (SrL) is a multifunctional hydrolase belonging to the SGNH family. Here site-directed mutagenesis (SDM) was used for the first time to investigate the functional significance of the conserved amino acid residues Ser10, Gly54, Asn82, Asn213, and His216 in the active site of SrL. The hydrolytic activity of SrL variants was determined using *para*-nitrophenyl (*p*NP) esters with C4, C8, and C16 fatty acid chains. Mutation of Ser10, Asn82, or His216, but not Gly54, to Ala abolished lipase activity for all substrates. In contrast, the Asn213Ala variant showed increased enzymatic activity for C8 and C16 *p*NP esters. Molecular dynamics (MD) simulations showed that the interactions between the long alkyl chain substrate (C16) and Ser10 and Asn82 were strongest in Asn213Ala SrL. In addition to Asn82, Gly54, and Ser10, several new constituents of the substrate binding site were recognized (Lys28, Ser53, Thr89, and Glu212), as well as strong electrostatic interactions between Lys28 and Glu212. In addition to the H bonds Ser10–His216 and His216–Ser214, Tyr11 interacted strongly with Ser10 and His216 in all complexes with an active enzyme form. A previously unknown strong H bond between the catalytically important Asn82 and Gly54 was uncovered, which stabilizes the substrate in an orientation suitable for the enzyme reaction.

## 1. Introduction

Streptomycetes are Gram-positive mycelial bacteria that possess remarkable capacity for the synthesis of bioactive compounds and a wide range of extracellular hydrolytic enzymes that they use to gain nutrients by degrading a complex organic material in their natural habitat [1,2]. Secreted enzymes that support the complex life cycle of *Streptomyces* spp. have been used in various industries and agriculture [3,4,5]. Specifically, applications of lipases from the genus *Streptomyces* are related to detergent, food, cosmetics, and pharmaceutical industries [5]. Recently, great interest has been attracted by the potential application of *Streptomyces* lipases in biofuel production due to their transesterification activity [3,6].

Extracellular lipase from *Streptomyces rimosus* (SrL) is among the best characterized streptomycetes’ lipolytic enzymes. This enzyme was first purified from the culture filtrate of a mutant of an industrial streptomycete, *S. rimosus* R6-554 W, and biochemically determined as a monomeric, basic protein that hydrolyzes triolein and *p*NP esters, preferring those of medium-size (C8–C12) acyl chain length [7]. The highest lipase activity was at 50–60 °C and in alkaline conditions (pH 9–10). Significant thermostability and pH stability was also the property of the isolated lipase from *S. rimosus*. Additional biochemical study showed that SrL possessed pronounced lipolytic activity toward various triacylglycerols and oils of vegetable and animal origin [8]. Glycerol esters of fatty acids with medium chain length (C8–C12) were hydrolyzed most efficiently by SrL where primary and secondary ester bonds were cleaved. An enzyme preference for the glycerol esters of unsaturated fatty acids over those of C16 and C18 saturated fatty acids was observed. SrL exerted also Tween-hydrolyzing activity with a rate comparable to that for hydrolysis of triacylglycerols and oils [8]. Furthermore, significant thioesterase and phospholipase activity of SrL was revealed [9]. Stability in organic solvent mixtures containing 50% ethanol, 1,4-dioxane, acetonitrile, or acetone was shown for SrL, which also catalyzed transesterification in *n*-hexane [8].

Amino acid sequencing of purified native SrL protein allowed the design of the primers and the cloning of the corresponding gene [10]. Sequence analysis revealed protein of 268 amino acid residues, including 34 amino acids of the signal peptide. In silico analysis of the deduced amino acid sequence established that SrL belongs to the GDS(L) subfamily of lipolytic enzymes of the family II [11], which have five conserved blocks (I–V) and four invariant, catalytically important residues: Ser, Gly, Asn, and His. The first consensus motif in block I, GDS(L), contains the putative active-site Ser and is located close to the N-terminus, different from the GxSxG motif found in many lipases situated near the center of the protein sequence. The recognition of four conserved residues (Ser, Gly, Asn, and His) in the GDS(L) subfamily led to a new designation of these enzymes as the SGNH-hydrolase superfamily [12].

Heterologous expression of SrL was obtained in *S. lividans* TK23 where maximal lipase activity was detected in culture filtrates of the late stationary phase [13]. The crystal structure of SrL was determined at the resolution of 1.75 Å [14,15]. The mature SrL protein comprises 234 amino acid residues and a three-layered αβα-sandwich fold typical of the SGNH-hydrolase superfamily formed by five parallel β-strands surrounded by 11 α-helices and by two 3_10_ helices. The 3D structure of SrL is stabilized by the three disulfide bonds and bears the closest similarity to that of the phospholipase A_1_ from *S. albidoflavus* (SaPLA1; 64.4% amino acid sequence identity) and the esterase from *S. scabies* (SsEst; 22% sequence identity). Indeed, the crystal structure of SrL was resolved by molecular replacement using a homology model based on the crystal structure of SaPLA1 [16]. In the SrL active site, analogous interactions to the ones in SsEst were observed, indicating that Ser10 and His216 were catalytic residues. Namely, earlier reports on the crystal structures of the free enzyme and enzyme-inhibitor complexes revealed that the *S. scabies* esterase active site contains a dyad of Ser14 and His283, which closely resemble the two components of typical Ser–His–Asp(Glu) triads of serine hydrolases, but lacks the carboxylic acid component [17]. These studies confirmed the nucleophilic role of Ser14 and identified the oxyanion hole in SsEst. Similar to SsEst, analysis of the crystal structure of SrL did not show any charged residue (i.e., a third residue of the typical lipase catalytic triad) in the vicinity of His216. SaPLA1 also possesses a catalytic dyad (Ser11–His218). An additional Ser (at position 216) is suggested to stabilize the imidazole moiety of catalytic His218 in SaPLA1.

While experimental data supported the crucial roles of Ser and His in the *S. scabies* esterase active site, those for SrL are missing. Since until now no crystal structure of SrL in complex with ligand (inhibitor or substrate) is resolved, experimental evidence or confirmation is missing regarding the functional importance of conserved amino acid residues indicated by structural analogy or by in silico studies [15,18]. To gain insight into the structure–function relationship of extracellular lipase from *S. rimosus*, this research used an experimental approach based on the site-directed mutagenesis and determination of the hydrolytic activity of purified enzyme variants for three substrates of different fatty acid chain lengths. In addition, MD simulations were used to investigate substrate binding for the wild-type (WT) and mutated variants of SrL.

## 2. Results

### 2.1. Selection of Mutations Predicted to Affect Enzyme Functionality

**Figure 1:**
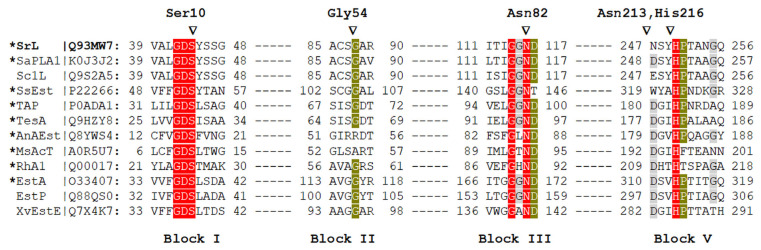
Multiple sequence alignment of selected protein sequences that belong to the GDS(L) family. Enzyme names and UniProtKB accession numbers are shown (SrL, extracellular lipase from *S. rimosus*; SaPLA1, phospholipase from *S. albidoflavus*; Sc1L, lipase from *Streptomyces coelicolor*; SsEst, esterase from *S. scabies*; TAP, thioesterase/protease/phospholipase from *Escherichia coli*; TesA, lysophospholipase from *Pseudomonas aeruginosa*; AnAEst, lipase from *Nostoc* sp.; MsAcT, arylesterase from *Mycolicibacterium smegmatis*; RhA1, rhamnogalacturonan acetylesterase from *Aspergillus aculeatus*; EstA, esterase from *Pseudomonas aeruginosa*; EstP, esterase from *P. putida*; XvEstE, esterase from *Xanthomonas vesicatoria*). The asterisk indicates an enzyme with a determined 3D structure. The conservation of marked amino acids in the alignment is color-coded (red, 100%; green, 80%; gray, 60%). Amino acids of SrL that were changed in this study are marked with triangles. The numbers next to the aa shown above the blocks indicate their position in mature SrL [10]. The alignment was constructed in MAFFT [20,21] using the G-INS-1 strategy and MAFFT homologs to improve the aligning of distantly related sequences.

Sequence analysis placed SrL into the SGNH-hydrolase superfamily (Pfam: CL0264) and GDSL-2 family (Pfam: PF13472) composed primarily of bacterial sequences [19]. The crystal structure determination revealed its similarity to the 3D structures of phospholipase A1 from *Streptomyces albidoflavus* and esterase from *Streptomyces scabies*. Several biochemically well-characterized and structurally resolved enzymes belonging to the GDSL-2 family were selected (Figure 1), and a multiple-sequence alignment was performed. Figure 1 shows the sequence conservation within the four blocks and the presence of catalytically important residues. To question the functional importance of conserved residues Ser10, Gly54, Asn82, and His216, belonging to blocks I, II, III, and V (Figure 1), lipase variants in which those residues were substituted by alanine were generated. Since SGNH hydrolases from genus *Streptomyces* (three out of four) did not have a highly conserved Asp in block V, additional lipase variants Asn213Ala and Asn213Asp were prepared to investigate whether the Asn213, a residue near the assumed catalytic His 216, influenced SrL activity.

### 2.2. The Impact of Targeted Amino Acid Mutations on Enzyme Functionality

**Figure 2:**
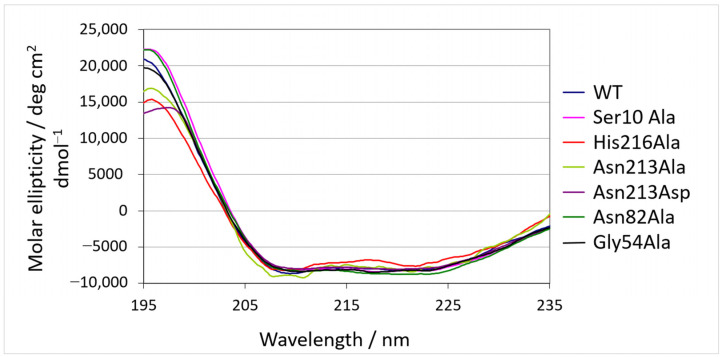
Circular dichroism spectra of SrL and its variants.

Recombinant SrL and its mutants were produced in *S. lividans* TK23 and purified to homogeneity as described in Section 4. First, it was analyzed whether the introduced mutations led to conformational changes in the SrL variants. Structural characteristics of purified enzyme variants together with WT lipase were evaluated by circular dichroism (CD). As shown in Figure 2, almost identical CD spectra were obtained for all proteins, suggesting that the introduced mutations did not lead to changes in the secondary structures of the proteins.

**Figure 3:**
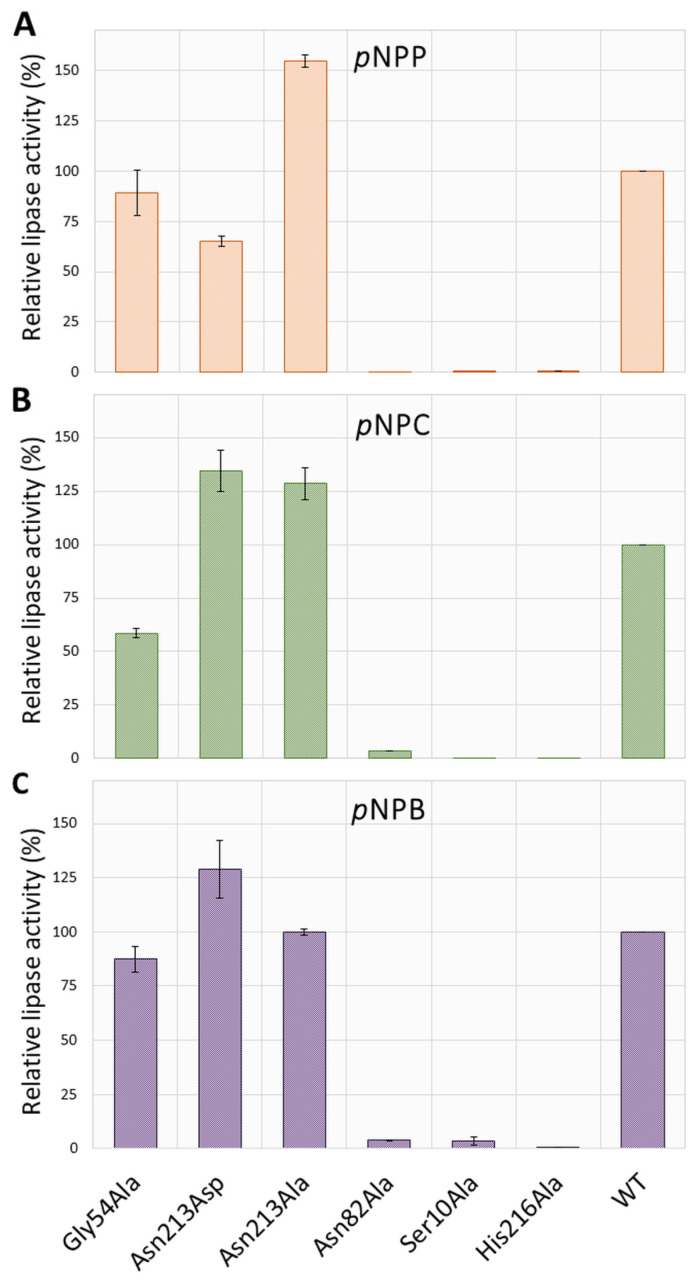
Comparison of the enzyme activities toward *p*NP esters with different acyl chain lengths (**A**–**C**). The activities are expressed relative to the specific activity of the wild type (100% = 797.40 U/mg protein for *p*NPC, 549.62 U/mg protein for *p*NPP, and 2.70 U/mg protein for *p*NPB) and represent the average of three measurements. Numerical values of standard deviations (SD) are given in Table S1.

Next, the specific activity of the purified enzymes was determined for three *p*NP esters with different fatty acid chain lengths: *p*-nitrophenyl butyrate (*p*NPB; C), *p*-nitrophenyl caprylate (*p*NPC; C8), and *p*-nitrophenyl palmitate (*p*NPP; C16). As shown in Figure 3, hydrolytic activity of Ser10Ala, Asn82Ala, and His216Ala was abolished for all examined substrates. The replacement of Gly54 with alanine residue decreased lipase activity with *p*NPC by about 40%, while the hydrolysis of *p*NPP and *p*NPB was almost unchanged (~10% decrease), compared with WT SrL. The substitution of Asn213 with Ala produced an enzyme variant with significantly higher activity for *p*NPP (~55%) and *p*NPC (~29%), while the activity toward *p*NPB remained unchanged in comparison with WT. The activity of mutant Asn213Asp was moderately enhanced toward *p*NPB (~29%) and *p*NPC (~34%) but decreased by ~35% with long acyl chain length substrate *p*NPP, compared with WT (Figure 3).

Out of the three tested substrates, *p*NPC was the preferred one in all active enzyme variants: the specific activities of WT, Asn213Ala, and Gly54Ala were 797, 1026, and 467 U/mg protein, respectively. Activity toward *p*NPP was somewhat lower for WT and Asn213Ala (31% and 17%, respectively), and that with *p*NPB was two orders of magnitude lower than the activity toward *p*NPC.

### 2.3. Biophysical Characterization

To evaluate the thermal stability of active lipases, differential scanning calorimetry (DSC) was used as the most direct method to obtain the melting temperature and enthalpy of the thermal unfolding process. As organic solvents are broadly utilized in biocatalysis, the thermal stability of WT enzyme and its active variants in buffer with the addition of 20% DMSO (*v*/*v*) was evaluated. The results are presented in Table 1, while baseline subtracted thermograms are shown in Figure S1. According to the obtained melting temperatures (Table 1), the WT is the most stable enzyme form (*T*_m_ 66.0 °C and 61.3 °C, without and with DMSO, respectively), followed by Asn213Asp, Gly54Ala, and Asn213Ala (*T*_m_ 62.1 °C and 54.0 °C, respectively). In terms of molar enthalpies of the unfolding process, there were no considerable differences between different active SrL variants. The same is true for the measurements in the buffer with 20% DMSO. Although the molar enthalpies are considerably lower (>80 kJ/mol), when the organic solvent is present, the different lipases show similar unfolding enthalpy values.

### 2.4. Molecular Simulations

In total, more than 5 µs of MD simulations were performed to investigate whether mutations lead to deformation of the protein conformation and to determine the binding mode of the long fatty acid chain substrate (C16) to SrL. Since the experimental 3D structure of an SrL–ligand complex has not yet been determined, the adaptive steered MD simulations (ASMD) were combined with MD simulations to determine the substrate binding to the active site of SrL.WT SrL and its variants Gly54Ala, Asn82Ala, and Asn213Ala were simulated both as free proteins and in complex with the long-chain substrate *p*NPP. The complexes were constructed using the atomic coordinates of ligand-free SrL and ASMD simulations as described in Section 4.

### 2.5. MD Simulations of Free Enzyme Forms

**Figure 4:**
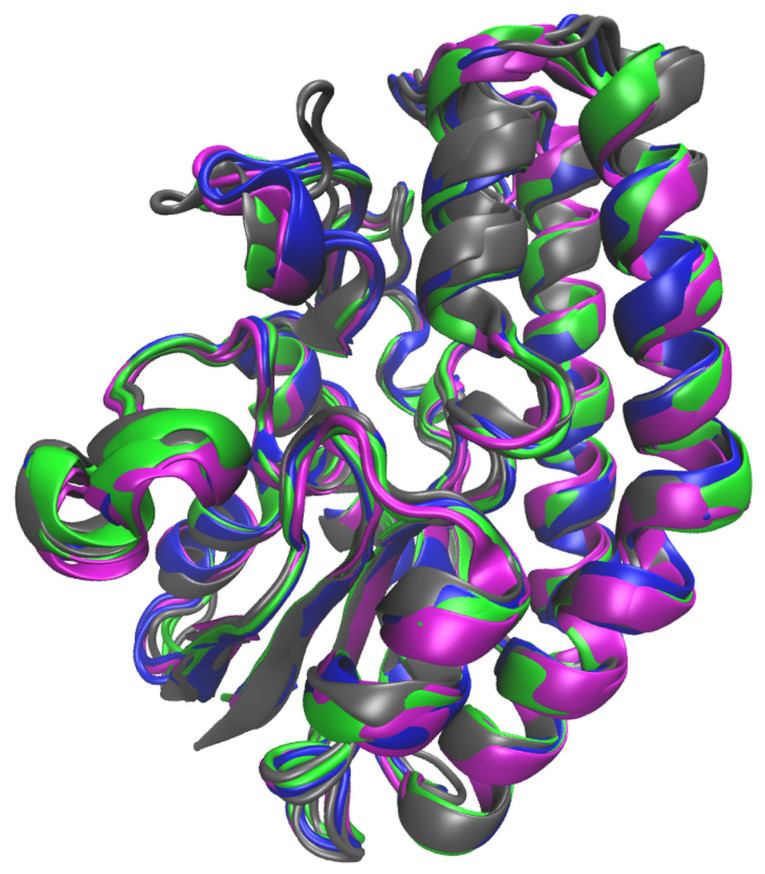
Overlay of the structures of WT SrL (colored magenta) and its mutants Asn213Ala (colored blue), Gly54Ala (colored gray), and Asn82Ala (colored green) obtained after 300 ns of MD simulations at 300 K in water (OPC water molecules) solution.

Figure 4 shows an overlay of the WT with the SrL variants, indicating that the mutations did not cause protein deformation. These results were in good agreement with the circular dichroism result (Figure 2). Also, during the 300 ns of MD simulations, the structure of WT SrL and its variants remained stable, and the RMSD values, which described the deviation of the protein structures sampled during the simulations from the equilibrated structure, were mostly ~1.0 Å (Figure S2). According to the MM/GBSA free energy calculations of the ligand-free lipase variants (Table S2), the WT and Gly54Ala variants were the most stable, and the Asn213Ala variant appeared to be the least stable variant during the 300 ns of MD simulations of the ligand-free proteins at 300 K. The Ser10–His216 distance was mostly in the range of 2–4 Å for all variants, although it was generally shorter for WT SrL and its Asn213Ala variant than for the Gly54Ala and Asn82Ala variants (Figure S3).

### 2.6. MD Simulations of the Lipase–Substrate Complex

**Figure 5:**
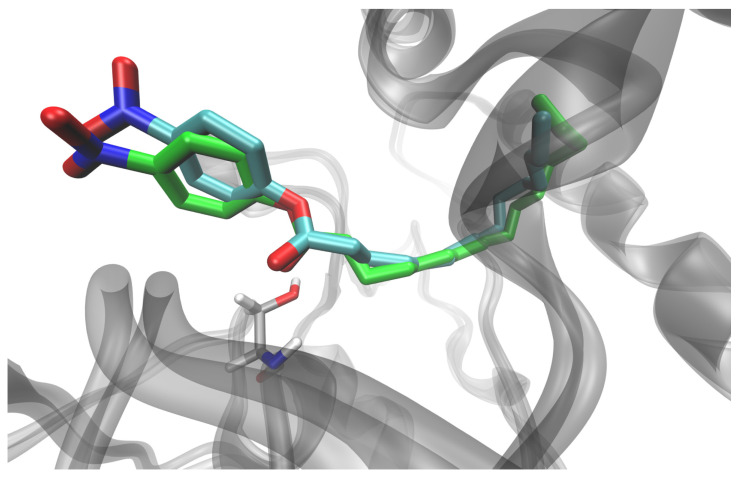
Orientation of the ligand in the SrL–*p*NPP complex, obtained by ASMD simulation (cyan), for the *p*NPP with alkyl chain accommodated in tunnel 1 and after additional 100 ns of unconstrained MD simulation of the solvated complex at 290 K (green). The position of Ser10 in the later structure is shown (light gray sticks).

The web tool CAVER 3.0 (CAVER Web—tool for the analysis of tunnels and channels in protein structures (https://caver.cz/, accessed on 17 August 2021) [22] was used to determine the catalytic cavity in WT SrL. In addition, ASMD simulations were used to determine the possible orientations of *p*NPP in the cavity (see Section 4 for details of the procedure). The ASMD simulations were carried out for two different initial structures, i.e., for two different positions of the *p*NPP. In one, *p*NPP was placed in tunnel 3 (cyan) with the tail of the alkyl chain solvated (ASMD-T3 set), and in the other, the alkyl chain of *p*NPP was placed in tunnel 1 (green) with the phenyl ring and NO_2_ group solvated (ASMD-T1 set) (Figure S4). The SrL–*p*NPP complexes obtained as a result of the ASMD simulations were visually examined, and it was found that the orientation of *p*NPP in the complex obtained starting from the structure accommodated in tunnel 1 was more suitable for hydrolysis than the *p*NPP orientation in the complexes obtained starting from the alkyl chain occupying tunnel 3. In both cases, the most suitable conformation of the ligand was in the complexes obtained with a puling step of 0.5 Å. These structures were further simulated for 100 ns at 290 K. During the simulation of the structure from the ASMD-T3 set, *p*NPP moved away from Ser10, and most of the alkyl chain remained solvated (Figure S5). On the other hand, *p*NPP from the ASMD-T1 set remained close to the initial position and was nicely accommodated in the SrL catalytic pocket (see Figure 5) during the 100 ns of unconstrained MD simulations. MM/GBSA binding free energies also revealed higher stability of the complex from the ASMD-T1 set than from the ASMD-T3 set (−65.6 ± 4.2 kcal/mol vs. −43.6 ± 4.3 kcal/mol).

**Figure 6:**
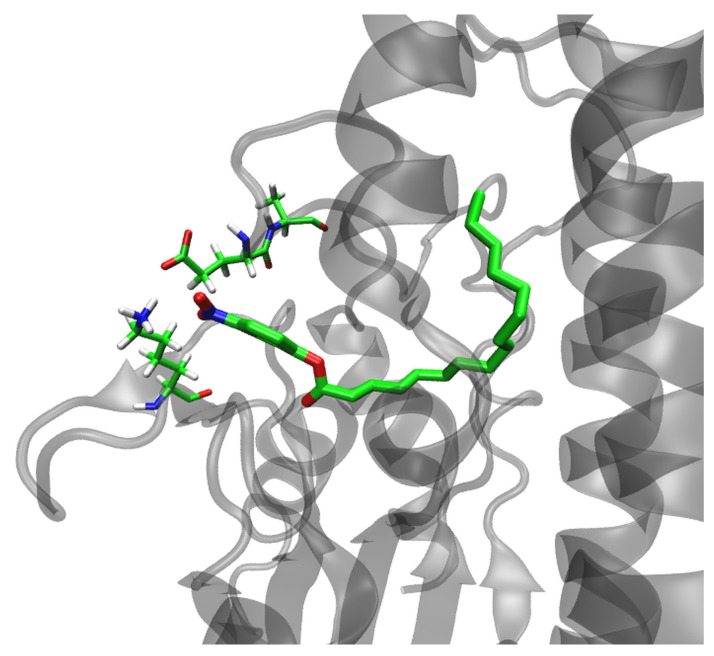
Interaction between Lys28 and Glu212 in the Asn213Ala–*p*NPP complex obtained after 300 ns of MD simulation in water (OPC water molecules) at 300 K.

Before further simulations, all structures were optimized and slowly heated (0 to 290 K), and the density of the system was equilibrated as described in Section 4. Three independent 200 ns long MD simulations were performed for each complex. With the exception of the Asn82Ala–*p*NPP complex, all other complexes were stable during each of the 200 ns of MD simulations. In the complex with N82A, *p*NPP migrated from the catalytic cleft into solvent after 77 ns in one simulation and after 55 ns in the other, so further simulations of this complex were not run. Based on the results, the stability of the complexes during the MD simulations, and the binding free energies calculated using the MM/GBSA [23] approach, as well as the distance between the C atom of the cleavable bond and Oγ of Ser10 (Table S3), the initial structures were selected for the following two independent, 300 ns MD simulations (for the WT complex and the complexes with the variants Gly54Ala and Asn213Ala). These complexes remained stable throughout the simulation period with RMSD values mostly below 1.5 Å (two independent 300 ns long MD simulations performed for each complex) (Figure S6), and *p*NPP remained close to its initial position (Figure S7). Interestingly, helices α6 and α11 approached each other (Figure S8), and the strong electrostatic interaction between Lys28 and Glu212 has been established in all variants (Figure 6) with geometry that often (>60% of the simulation time) fulfilled the conditions for hydrogen bonding. Moreover, these two residues interacted with *p*NPP.

**Figure 7:**
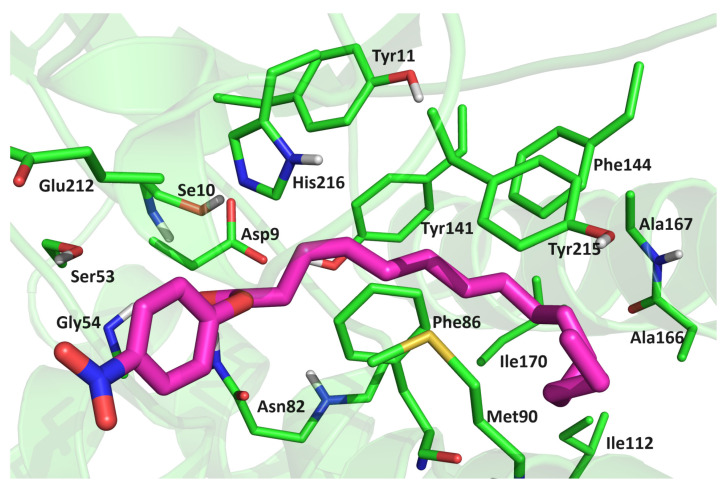
Position of *p*NPP in the Asn213Ala-*p*NPP complex after 300 ns of MD simulation in water (OPC-water molecules) at 300 K. The amino acid residues that have close contacts (distance less than 4 Å) with *p*NPP are indicated.

To evaluate the substrate binding affinities, the MM/GBSA, MM/PBSA [24], and LIE energies (Table 2 and Tables S4 and S5) were calculated. Although the calculations predicted similar binding affinities for all variants, the LIE method [25] revealed a slightly higher affinity of *p*NPP for the WT protein and MM/GBSA for the Asn213Ala mutant. In this stable binding mode, the hydrophilic ester moiety (O=C–O–Phe–NO_2_) of *p*NPP interacted with Ser10, Lys28, Ser53, Gly54, Asn82 (side chain), and His216, and occasionally with Glu212, while the hydrophobic, alkyl chain interacted strongly with Tyr11, Gly81, Asn82 (backbone), Ala84, Gly54, Phe86, Ala108 Ala167, Tyr141, Phe144, and Ile163. Significant stabilization was also achieved by Thr89 in the complexes with the WT protein and in the complexes with the mutants Gly54Ala and Asn213Ala. The alkyl chain was additionally stabilized by Met90 in mutant variants (Figures S9 and S10 and Table 3). The strongest interaction between *p*NPP and protein was achieved via the *p*NPP(O2)–Ser10(OH) hydrogen bond (Table 3 and Figure S11). In addition to Ser10, *p*NPP interacted strongly with Ser53, Gly54, Asn82, and Tyr141, forming either H bonds or strong electrostatic interactions with them during the simulation of all complexes (Table 3). With Phe86, Tyr141, and Phe144, the substrate had CH–π interactions during most of the simulation time (Figure 7), and with the other amino acids mentioned above, the substrate underwent electrostatic and van der Waals interactions that occasionally became hydrogen bonds.

Throughout the simulation, Ser10 and His216 were properly oriented and formed strong hydrogen bonds in all complexes. Interestingly, Tyr11, which was involved in stabilizing the substrate, also interacted strongly with Ser10 and His216 (Figure S12).

## 3. Discussion

The SGNH superfamily encompasses a large group of phylogenetically broadly distributed enzymes in all domains of life. The GDSL-like lipases/acylhydrolases are the most abundant in this superfamily. Most members of this group belong to bacteria, followed by plants and fungi [19]. Although some of them exhibit multifunctional properties, many members have not been functionally investigated, such as in *Arabidopsis*, where more than 100 GDSL lipases are recognized [12,26,27].

The native extracellular SGNH/GDSL lipase of *S. rimosus* was previously purified and biochemically characterized, the corresponding gene was cloned, and a high level of heterologous expression was obtained [7,8,10,13]. In the present study, site-directed mutagenesis was applied for the first time to investigate the function of amino acid residues suggested to be important for its hydrolytic activity. The assumption of catalytic relevance of Ser10, Gly54, Asn82, and His216 resulted from the primary structure analysis, which placed SrL in the SGNH hydrolase superfamily, and from the comparison of the crystal structure of ligand-free SrL [15] with similar 3D structures of two SGNH-hydrolyses (esterase from *S. scabies* and phospholipase from *S. albidoflavus*) [16,17].

Our results provide experimental evidence for the crucial importance of Ser10, Asn82, and His216 for the hydrolytic activity of SrL (Figure 3) as their substitution with Ala yielded inactive enzyme variants. On the other hand, point mutations (Gly54 to Ala and Asn213 to Ala) did not change the substrate selectivity of SrL. All active mutants preferred the C8 acyl chain length in *p*NP esters.

The activity of the His-tagged WT SrL enzyme was in very good agreement with previously reported specific activity of native SrL for *p*NPP (659 U/mg) and *p*NPC (850 U/mg) [7]. Replacement of Gly54, a conserved constituent of the block II in the SGNH family enzymes, with alanine caused only a slight to moderate decrease (depending on the *p*NP substrate) in the activity of the variant Gly54Ala compared with the WT enzyme. The GDSL protein family has been reported to contain a significant proportion of sequences with distinct variations in motifs that sometimes lack catalytically important residues. Such mutations can generate pseudoenzymes or enzymes with new properties [28,29]. Natural variations of conserved Gly to Ala and Arg have been reported for two members of the SGNH superfamily (Figure 1), acyltransferase from *Mycobacterium smegmatis* [30] recently reclassified as *Mycolicibacterium smegmatis* [31] and a serine hydrolase from cyanobacterium *Anabaena* sp. PCC 7120, respectively. By site-directed mutagenesis it was shown that Arg54 in *Anabaena* hydrolase has a role in substrate binding and catalytic activity [32].

In lipases with the catalytic triad Ser–Asp–His, the catalytic Asp is located at the position of Asn213 of WT SrL (block V); therefore, a protein mutated at this position was generated and analyzed. The SrL variant Asn213Asp showed slightly higher activity with *p*NPB and *p*NPC but lower activity with *p*NPP compared with the WT enzyme (Figure 3). On the other hand, the replacement of Asn213 with Ala improved the hydrolytic activity toward the medium- and long-chain substrates *p*NPC and *p*NPP. Although Asn213 did not interact directly with *p*NPP, it interacted with Lys28 and, together with Ser214, with the catalytic His216 (hydrogen bonds and electrostatically) in the MD simulations. Asn213 and Lys28 were located at turns (unstructured regions of SrL) that faced each other and separated the catalytic part of the substrate binding pocket from the surrounding solvent. The interactions between Lys28 and Asn213 and Glu212 consolidated and shaped the catalytic site of SrL and ensured proper alignment of the substrate through the hydrogen bonding network (in conjunction with strong electrostatic interactions) with the amino acid residues of the binding site.

MD simulations showed that the interaction between *p*NPP and the catalytic residue Ser10, as well as with Asn82 from the oxyanion hole, was stronger in the complex of *p*NPP with Asn213Ala than in the complex with the wild-type protein.

Molecular dynamics simulations revealed the key interactions between lipase and the long-chain *p*NP ester substrate, including H bonds and electrostatic interactions with Asn82 and H bonds with Gly54 and Ser10. The observed stabilization of the substrate carbonyl oxygen with Asn82 and Gly54 suggested that they acted as oxyanion hole residues, stabilizing the negatively charged tetrahedral intermediates during the hydrolytic reaction. Moreover, Asn82 (NH_2_ group from the side chain) formed a strong H bond with the carbonyl oxygen of Gly54 and stabilized it in the orientation suitable for the enzymatic reaction. In addition, stable H-bonding between His216 and Ser214 was observed during the MD simulations. Overall, our computational results emphasized the crucial importance of Ser10, Asn82, and His216 for the catalytic activity of SrL, which was consistent with the experimental analysis of SrL variants. The obtained results supported the hypothesis of a variation in the active site of *S. rimosus* lipase [15], consisting of Ser10 and His216 but lacking the Asp (or Glu) residue that ensured the correct orientation of the imidazole ring of His in typical serine hydrolases. Such variation has been reported for the two SGNH hydrolases structurally closest to SrL, *S. scabies* esterase and *S. albidoflavus* phospholipase A1 [16,17]. In addition, the molecular modeling results of our study showed previously unreported interactions occurring within the active site of SrL lipase in the presence of substrate, thus providing a good basis for future research on this interesting class of SGNH enzymes.

## 4. Materials and Methods

### 4.1. Bacterial Strains and Cultivation Conditions

The bacterial strains and culture media used in this study are listed in Table S6. *E. coli* was cultivated at 37 °C in LB medium [33]. *Streptomyces lividans* TK23 was grown at 30 °C in liquid CRM medium [34] for plasmid isolation or inoculum preparation, solid MS medium for sporulation, solid R5 medium for protoplast regeneration, and liquid GR_2_d medium for lipase production, as described previously [35,36]. When appropriate, antibiotics were added to the media in the following final concentrations: 100 mg/mL ampicillin, 25 mg/mL thiostreptone, and 10 mg/mL kanamycin (Sigma-Aldrich, St. Louis, MO, USA). Only to MS medium thiostreptone was added at a concentration of 50 mg/mL.

### 4.2. Cloning and Site-Directed Mutagenesis

The wild-type *srl* gene was amplified by PCR using forward primer srlEco, reverse primer srlHind (Table S7), and plasmid pDJ5 as a template ([10], Table S8) according to the procedure detailed in Supplementary Materials. The amplified 840 bp DNA fragment was ligated into the pGEM-T vector (Promega, Madison, WI, USA), which was used to transform *E. coli* XL1 cells. The recombinant plasmid, SrLpGEM, was purified from selected transformants with a QIAprep Spin Miniprep Kit (Qiagen, Hilden, Germany), and the sequence of the lipase gene was verified by sequencing. The plasmid, SrLpGEM, was used either to clone the *srl* gene into the bifunctional vector pANT849pWB19N [35] or as a template to generate targeted mutations in the lipase gene using the site-directed mutagenesis method [37]. Modifications introduced to this protocol are described in detail in Supplementary Materials. The obtained SrLpGEM constructs and pANT849pWB19N [38] were used for the transformation of methylation-deficient *E. coli* GM119. Non-methylated plasmids were digested with *Eco*RI and *Hin*dIII (Fermentas, Waltham, MA, USA), and DNA fragments (5300 bp pANT849 and 840 bp *srl* gene or its mutated variants) were gel purified (QIAquick Gel Extraction Kit, Qiagen, Hilden, Germany) and ligated using T4 DNA ligase (Fermentas, Waltham, MA, USA). The ligation mixture was used to transform *Streptomyces lividans* TK23 protoplasts, as described [35,36]. The recombinant plasmids, SrLpANT, carrying the *srl* gene or its variants were purified from selected transformants by an alkaline lysis procedure [36], and the presence of the *srl* insert was confirmed by PCR and sequencing.

### 4.3. Biosynthesis and Purification of SrL Lipase Variants

The purification of His-tagged SrL lipase variants was performed according to the protocol developed previously [35], which includes (i) growth of *S. lividans*-SrLpANT in GR_2_d liquid medium for ~6 days or until the extracellular lipase in the culture supernatant showed maximum activity toward *p*NPP (Sigma-Aldrich, St. Louis, MO, USA), (ii) precipitation of extracellular proteins with 80% ammonium sulfate and subsequent dialysis, and (iii) purification of His-tagged lipases by Ni-NTA Agarose gravity flow chromatography (Qiagen, Hilden, Germany). The only change compared with the described method was the increase in imidazole (30 mM) in the washing step, which allowed the removal of non-specifically bound proteins, thus avoiding gel filtration in the purification protocol. The purified proteins were analyzed by SDS-PAGE, and their concentration was determined using NanoDrop 2000 (Thermo Fisher Scientific, Fermentas, Waltham, MA, USA).

### 4.4. Circular Dichroism (CD) Spectrometry

Circular dichroism (CD) spectra of purified lipase variants were recorded in a range of 235 to 195 nm by using a J-715 Spectropolarimeter (JASCO, Tokyo, Japan) at room temperature. The concentration of the samples was ~0.5 mg/mL (in 10 mM phosphate buffer pH 6.8, 0.2 M NaCl).

### 4.5. Enzyme Activities

Lipase activity was measured spectrophotometrically at room temperature (22 °C) by monitoring the release of *p*NP from three different substrates: *p*NPP, *p*NPC, and *p*NPB. Enzymatic reaction with buffered *p*NPP or *p*NPC emulsion was performed as described previously [35]. The reaction mixture was created by mixing a substrate stock solution in dioxane and buffer (50 mM Tris-HCl pH 8, 5 mM sodium deoxycholate) to a final substrate concentration of 0.33 mM *p*NPP or 0.36 mM *p*NPC and 2.5% dioxane (*v*/*v*). The hydrolysis of *p*NP esters was initiated by adding 10 µL of the enzyme (0.01 mg/mL) into 1 mL of the reaction mixture. The catalytic activity of lipase toward *p*NPP and *p*NPC was measured at 410 nm by UV/Visible Varian Cary 100 Bio spectrophotometer. The spontaneous hydrolysis of each of the substrates was also examined. A similar protocol was used for *p*NPB, except that the hydrolysis was carried out at pH 7.2. The substrate concentration in the reaction mixture was 1 mM, and the dioxane concentration was 1% (*v*/*v*). Enzyme reactions were started by adding 10 µL of 1 mg/mL enzyme into a 1 mL reaction mixture. One unit (1U) of lipase activity was defined as the amount of enzyme needed to liberate 1 µmol of *p*NP per minute at the assay conditions.

### 4.6. Enzyme Stability Measurements

Thermal stability measurements of SrL and its active variants (Asn213Ala, Asn213Asp, and Gly54Ala) were completed in a Nano-DSC (TA Instruments, Waters Corporation, New Castle, DE, USA) differential scanning microcalorimeter. Samples were prepared by diluting the purified protein to a concentration of 1 mg/mL in buffer (10 mM phosphate buffer, 200 mM NaCl, pH 8) and buffer/DMSO mixtures (20% DMSO, *v*/*v*), followed by degassing for 15 min on a degassing station (TA Instruments, Waters Corporation, New Castle, DE, USA). The working volume of the calorimeter was 300 µL, and the thermal cycle was performed with a 60 s equilibration time in the temperature range of 10–90 °C at a scan rate of 1 °C/min. The buffer–buffer and buffer/20% DMSO–buffer/20% DMSO thermal scans were subtracted from the protein measurements. The results were assessed with Nano Analyze software, 3.11.0 (TA Instruments, Waters Corporation, New Castle, DE, USA), and thermal unfolding characteristics (*T*_m_—melting temperature, ∆*H*—enthalpy) were evaluated.

### 4.7. Selection of the Crystallographically Determined Structure of SrL for Molecular Modeling and Parameterization of the System

The crystallographically determined structure of SrL, PDB_id 5MAL, was used as the initial structure for our molecular modeling study. The asymmetric unit comprised two monomeric SrL molecules, A and B, which did not reveal significant conformational differences: the secondary structural elements overlapped well (RMSD of 0.54 Å). For the molecular modeling study, molecule A was utilized. The structure showed two possible orientations of the amino acid residues Ser129, Gln135, and Glu212. Orientation A was chosen for Ser129 and Gln135 because in this orientation they had more stable interactions with the rest of the protein, whereas for Glu212 both orientations were studied, orientation A with Cα-Cβ-Cγ-Cσ equal to −96.5° and orientation B with Cα-Cβ-Cγ-Cσ equal to −63.7°.

The SrL structure had six Cys residues all connected by disulfide bonds. In the model, all Arg and Lys residues were positively charged (+1e), while Glu and Asp residues were negatively charged (1e), as expected under physiological (experimental) conditions. According to their ability to form hydrogen bonds with surrounding residues, His residues 42, 195, and 216 were protonated at Nδ, and His181 and His205 were protonated at Nε. Parameterization was performed using the FF19SB force field [39] for protein and the compatible GAFF2 force field [40] for ligand. The AM1-BCC atomic charges [41] were assigned to the ligand. The mutated proteins, Gly54Ala, Asn82Ala, and Asn213Ala, were prepared using tleap, a basic preparation program for Amber simulations available within the AMBER20 package (http://ambermd.org, accessed on 20 August 2021). Proteins and protein–substrate complexes were neutralized with Na^+^ ions and solvated in an octahedral box filled with OPC water molecules [42] recommended for use with the ff19SB force field. The minimum distance between the solvated complex and the edge of the box was 11 Å. The solvated systems were minimized, followed by heating, density equalization, and productive MD simulations.

### 4.8. MD Simulations Details

All simulations were carried out using the AMBER20 suite of programs [43,44]. The minimization of the prepared systems (ligand-free protein variants and their complexes with *p*NPP) was performed in three cycles. In the first cycle of optimization (1500 steps), water molecules were relaxed, while the rest of the system was harmonically restrained with a force constant of 32 kcal mol^−1^ Å^−2^; in the second cycle (3500 steps), the protein backbone atoms were restrained with a force constant of 12 kcal mol^−1^ Å^−2^; and in the third cycle (2500 steps), the system was completely relaxed. The energy-optimized system was heated in three steps (from 0 to 100 K, from 100 K to 200 K, and from 200 K to 300 K) (each interval 10 ps) using the NVT ensemble and the time step of 0.5 fs, followed by 3 ns of density equilibration (NPT ensemble and the time step of 1 fs). The two independent MD simulations of 300 ns duration were performed for the ligand-free protein variants, and for their complexes with *p*NPP, obtained by Docking, three independent MD simulations (each 200 ns long) were performed (see below). For each complex of the variant the most stable structure obtained during these 200 ns long MD simulations was selected, and its two replicas were simulated for an additional 300 ns. During productive MD simulations, the time step was 2 fs, and the SHAKE algorithm was used to constrain covalent bonds with hydrogen atoms. The pressure was maintained at 1 atm with a pressure relaxation time of 1 ps using the Berendsen barostat [45], while the system temperature was kept constant at 300 K with a collision frequency of 5 ps^−1^, using the Langevin thermostat [46]. Simulations were performed using periodic boundary conditions (PBCs) with a cutoff of 11 Å, while the particle mesh Ewald (PME) method was used to calculate long-range electrostatic interactions.

### 4.9. Elucidation of the Binding of pNPP to SrL Using Adaptive Steered MD Simulations (ASMD)

For binding of the substrate (*p*NPP) to the active site of lipase, we performed ASMD simulations. In ASMD, an external force is applied to the selected reaction coordinate to allow the system to move in the desired direction during simulations of MD [47,48]. The average nonequilibrium work exerted on the system during ASMD is referred to as the potential of mean force (PMF) and, according to the Jarzynski equation, reflects the relative free energy of binding (∆G) [49]. ASMD simulations are useful for investigating possible pathways for ligand binding and release to macromolecules but can also be used to tune ligand binding into the active site of an enzyme if the active site is known.

Experimental study on SrL combined with previous computational studies on SrL showed that Ser10 and His216 make the catalytic dyad responsible for ester hydrolysis in SrL and that acyl chains most probably bind in the hydrophobic tunnel and in the hydrophobic “gorge” [15,18]. The starting point for our ASMD simulations was a fully solvated system consisting of the optimized and equilibrated structure of the ligand-free protein variant with the ligand (*p*NPP) in the extended conformation manually placed in the hydrophobic pocket with the C atom of the scissile C-O(R) bond about 6 Å away from Ser10. Three series of ASMD simulations were performed; in one of them, the velocity of the substrate pooling into the proposed binding funnel was 2.1 Å/ns, and in two ASMD simulations it was 0.5 Å/ns (during the simulations, C16 was pooled toward Oγ(Ser10)). Each of the ASMD simulations was performed in seven steps.

The obtained complexes were minimized, heated, and density equilibrated, and three independent 200 ns long productive MD simulations were performed for each complex (a detailed description of the procedure is given in Supplementary Materials). The ASMD simulations as the following productive MD simulations were performed using the TIP3P water model. The structure for which the lowest binding free energy was determined was used as a starting point for 300 ns long MD simulation with OPC water molecules. For this purpose, the system was reparameterized using the OPC water box, and after minimization and equilibration (the procedure equivalent to that used for the ligand-free enzyme), a 300 ns long MD productive simulation was performed.

### 4.10. MM/PBSA and MM/GBSA Calculations

The substrate binding MM/PBSA and MM/GBSA energies were obtained using the MM/PBSA py script as implemented in the AMBER20 program [43]. MM/GBSA calculations were performed using “igb = 5” [23]. MM/GBSA energies were calculated on 20 ns long intervals sampled throughout the trajectories sampled during three independent 200 ns long productive MD simulations. For the final trajectory obtained during 300 ns of productive MD simulations, both MM/GBSA and the MM/PBSA [24] energies were calculated for the structures sampled during the intervals 80–100 ns, 180–200 ns, 280–300 ns, 100–300 ns, and 1–300 ns using the single-trajectory approach. To test the influence of the frequency of sampling on the results, we performed a few calculations using both 100 and 1000 structures. Since the difference was negligible, all other calculations were performed using 100 structures and, in the case of the 1–300 ns interval, 150 structures. The MM/PBSA calculations were performed using a salt concentration of 0.1 M. The dielectric constants were 80.0 for the solvent (water) and 2.0 for the solute. The polar component of the enthalpy of solvation was calculated by the Poisson–Boltzmann method, and the nonpolar component was determined by ΔH_nonpol_ = γSASA + β, where the solvent accessible surface area (SASA) was calculated using the MolSurf program [50].

### 4.11. Data Analysis

To trace the conformational space spanned by the SrL structures during the MD simulations and to determine the amino acid residues that most strongly stabilized the binding of *p*NPP, a series of analyses were performed. Detailed analyses of the geometry, the population of intermolecular hydrogen bonds, and LIE calculations (linear interaction energy approach introduced by Åqvist at al. [25]) were performed using the CPPTRAJ module of the AmberTools program package [43]. LIE calculations were performed using cutoff values 9 Å and 11 Å, for Van der Waals and electrostatic energy calculations.

## 5. Conclusions

The site-directed mutagenesis, performed for the first time on conserved residues in the active site of the SGNH lipase from *S. rimosus*, confirmed the essential importance of Ser10, Asn82, and His216, while Gly54 could be substituted by Ala without a significant decrease in hydrolytic activity. The replacement of Asn213 with Ala increased SrL activity, suggesting that SDM can improve the function of this enzyme. MD simulations identified the key interactions between the lipase and the long-chain substrate (*p*NPP). In addition, a previously unrecognized H bond Asn82–Gly54 and a strong electrostatic interaction between Lys28 and Glu212 were uncovered in the enzyme–substrate complexes, the significance of which should be investigated for catalysis.

## Figures and Tables

**Table 1:** Melting temperatures (*T*_m_) and molar enthalpy (∆_r_*H*) of the thermal unfolding process for WT and enzyme variants: Asn213Ala, Asn213Asp, and Gly54Ala. All proteins were measured in phosphate buffer and buffer/DMSO mixture.

Buffer (10 mM Phosphate, 200 mM NaCl, pH 8)				
SrL	*c* (mg/mL)	*M*_w_ (kDa)	*T*_m_ (°C)	∆_r_*H* (kJ/mol)
WT	1	25	66.0	534.8
Asn213Ala	1	25	62.1	534.8
Asn213Asp	1	25	63.9	537.6
Gly54Ala	1	25	63.0	535.1
**Buffer/DMSO (10 mM Phosphate, 200 mM NaCl, pH 8, 20% DMSO, *v*/*v*)**				
WT	1	25	61.3	456.7
Asn213Ala	1	25	54.0	450.5
Asn213Asp	1	25	60.0	452.1
Gly54Ala	1	25	58.5	449.9

**Table 2:** LIE, MM/GBSA, and MM/PBSA free energy approximations for the binding of *p*NPP to WT SrL and its single-point mutants Gly54Ala and Asn213Ala. The energies (kcal/mol) and standard deviations were calculated for the sets of conformers sampled throughout the 300 ns and during the last 100 ns of the MD simulations in water (OPC water molecules) at 300 K. MM/GBSA and MM/PBSA energies were calculated for ionic strengths of 0 (100–300 ns) and 0.1 M at smaller intervals and for the entire 300 ns, respectively. Values obtained by averaging values energies calculated on structures sampled during two independent simulations of the same system are shown in bold.

SrL, Variants and Run	Method				
	LIE	MM/GBSA (1–300 ns)	MM/PBSA (1–300 ns)	MM/GBSA (200–300 ns)	MM/PBSA (200–300 ns)
WT-1	−11.9	−67.5 ± 4.2	−26.7 ± 3.7	−68.0 ± 3.3	−27.2 ± 4.1
WT-2	−13.7	−67.6 ± 4.2	−29.0 ± 4.1	−66.2 ± 3.6	−28.3 ± 4.2
WT-average	**−12.8**	**−67.6**	**−27.9**		
Asn213Ala-1	−11.8	−69.5 ± 3.3	−28.2 ± 3.3	−68.9 ± 3.4	−28.2 ± 3.7
Asn213Ala-2	−12.0	−68.9 ± 3.5	−27.2 ± 3.4	−69.5 ± 3.3	−27.1 ± 3.5
Asn213Ala-average	**−11.9**	**−69.2**	**−27.7**		
Gly54Ala-1	−11.3	−67.8 ± 3.2	−27.7 ± 3.0	−68.5 ± 3.4	−27.5 ± 3.0
Gly54Ala-2	−11.6	−67.1 ± 3.6	−26.9 ± 3.3	−68.5 ± 3.4	−26.4 ± 3.1
Gly54Ala-average	**−11.5**	**−67.4**	**−27.3**		

**Table 3:** Intermolecular, *p*NPP—SrL hydrogen bonds during MD simulations of the complexes with WT SrL and its mutants Asn213Ala and Gly54Ala. Indicated is the percentage of simulation time during which a particular hydrogen bond exists. In cases where an amino acid residue interacts with the substrate through several different H bonds, the percentage of its population may exceed 100. The results are for the simulations are performed at 300 K solvated in OPC water.

	HB Population					
AA/Variant	WT-1	WT-2	Asn213Ala-1	Asn213Ala-2	Gly54Ala-1	Gly54Ala-2
Ser10	179	196	184	202	153	196
Tyr11	27	27	8	10	6	11
Lys28	18	71	-	-	-	-
Ser53	67	-	86	65	52	55
Gly54	30	52	31	26	-	-
Asn82	55	19	88	88	68	90
Thr89	27	56	-	-	-	-
Met90	-	-	42	39	17	24
Tyr141	44	20	38	25	25	24

## Data Availability

Data is contained within the article and supplementary material.

## Supplementary Materials

The supporting information can be downloaded at https://www.mdpi.com/article/10.3390/ijms25010595/s1.
