# Supplementary material for: Evaluation of the Structure–Function Relationship of SGNH Lipase from Streptomyces rimosus by Site-Directed Mutagenesis and Computational Approach
Source: Int J Mol Sci. 2024 Jan 2;25(1):595. doi: 10.3390/ijms25010595 (PMC10779480; doi:10.3390/ijms25010595)
Supplement: Supplementary file 1 [file ijms-25-00595-s001.zip › ijms-2727980-supplementary.pdf]

SUPPLEMENTARY MATERIAL OF THE MANUSCRIPT

Extracellular SGNH lipase from *Streptomyces rimosus*: structure-function study by experimental and computational approach

Želimira Filić [1,‡], Ana Bielen [2,‡], Ela Šarić [1], Mirsada Ćehić [1,§], Ivo Crnolatac [3], Sanja Tomić [3,*], Dušica Vujaklija [1,*] and Marija Abramić [3]

Supplementary results

Supplementary Tables

Table S1. Normalized values of enzyme activities toward *p*NP esters with different acyl chain length presented in **Figure 3** in the main text.

	**Normalized activity ± standard deviation (%) of SrL and its mutated variants toward different substrates**		
**SrL variant**	***p*NPP**	***p*NPC**	***p*NPB**
Gly54Ala	89.15 ± 11.23	58.55 ± 2.10	87.39 ± 5.85
Asn213Asp	64.85 ± 2.56	134.39 ± 9.75	128.97 ± 13.29
Asn213Ala	154.51 ± 3.14	128.55 ± 7.49	99.90 ± 1.58
Asn82Ala	-0.08 ± 0.01	3.57 ± 0.01	3.77 ± 0.12
Ser10Ala	0.19 ± 0.01	-0.22 ± 0.02	3.27 ± 1.90
His216Ala	0.40 ± 0.01	-0.05 ± 0.01	0.46 ± 0.01
WT	100.00 ± 0.00	100.00 ± 0.00	100.00 ± 0.00

Table S2. The MM/GBSA estimate of the relative stability of the different variants (kcal/mol). Absolute values and standard deviations were calculated for the sets of conformers sampled during the last 100 ns of a 300 ns long MD (ionic strength = 0.1 M). The next table shows the mean value obtained by averaging for two independent simulations.

SrL variant	280-300 ns	100-300 ns
WT	-5838.4±43.7	-5839.8±45.3
WT-r2	-5863.3±41.7	**-5848.3±45.3**
Asn213Ala	-5767.0±44.3	**-5775.2±41.6**
Asn213Ala-r2	-5754.2±42.2	-5744.0±45.7
Gly54Ala	-5808.7±39.2	-5817.5±41.1
Gly54Ala-r2	-5831.4±47.4	**-5831.7±43.5**
Asn82Ala	-5771.2±40.2	-5766.1±40.1
Asn82Ala-r2	-5777.6±48.7	**-5772.8±42.4**

variant	100-300 ns
WT	-5844
Asn213Ala	-5760
Gly54Ala	-5825
Asn82Ala	-5769

Table S3. Distance between the the hydroxyl oxygen (Oγ)of the catalytic serine (S10) and the carbonyl carbon (C) of the substrate at the end of MD simulation and the lowest MM/GBSA energy calculated for the set of structures from the 10 ns long intervals sampled during the last 100 ns of MD simulations. The standard deviation (SD) is about 3 kcal/mol in all cases.

[*] The lowest MM/GBSA energy calculated during the last 100 ns of MD simulations.

**variant**	< Oγ (Ser10)-C(*p*NPP)>200 ns (Å)	Time of simulations	Simulation temperature	MM/GBSA* (kcal/mol)
**WT**	3.1	200 ns	290 K	-71
	3.1	200 ns	290 K	-65
	3.3	200 ns	300 K	-65
**Asn213Ala**	3.3	200 ns	290 K	-64
	3.3	200 ns	290 K	-71
	3.2	200 ns	300 K	-69
**Gly54Ala**	3.4	200 ns	290 K	-63
	3.3	200 ns	290 K	-66
	3.2	200 ns	300 K	-69
**Asn82Ala**	~10[#]	~ 77 ns	290 K	-47[#]
	~5.0[#]	~ 55 ns	290 K	-65[#]

[#] The substrate moved from its initial position and was more exposed to the solvent.

Table S4. The MM/GBSA aproximation of the binding free energy for *p*NPP binding to the wild type of SrL and its single point mutants. The energies (kcal/mol) and standard deviations are calculated for the sets of conformers sampled during 20 ns and 100 ns long intervals as specified. Ionic strenght was 0 M.

variant	80-100 ns	180-200 ns	100-200 ns	280-300 ns	200-300 ns	<100-300> and <1-300> ns *
WT-1	-64.9±3.1	-68.5±3.3	-68.5±3.3	-68.9±3.5	-68.0±3.3	-66.2±4.3<br>-67.5±4.2
WT-2	-69.1±4.2	-69.7±3.7	-64.9±3.3	-68.7±4.1	-66.2±3.6	-66.7±4.3<br>-67.6±4.2
Aan213Ala-1	-70.6±3.1	-69.4±3.6	-69.5±3.5	-68.1±2.9	-68.9±3.4	-69.2±3.4<br>-69.5±3.3
Asn213Ala-2	-67.2±3.5	-69.0±3.5	-68.4±3.6	-69.8±3.3	-69.5±3.3	-68.9±3.5<br>-68.9±3.5
Gly54Ala-1	-67.3±3.4	-67.1±2.8	-66.8±2.9	-69.8±3.2	-68.5±3.4	-67.7±3.3<br>-67.8±3.2
Gly54Ala-2	-69.1±3.5	-65.9±3.0	-66.2±2.9	-68.1±3.3	-68.5±3.4	-66.2±3.3<br>-67.1±3.6

*Averaged over the whole trajectory

Table S5. The MM/PBSA aproximation of the binding free energy for *p*NPP binding to the wild type of SrL and its single point mutants. The energies (kcal/mol) and standard deviations are calculated for the sets of conformers sampled during 20 ns and 100 ns long intervals as specified. Ionic strenght was 0.1.

variant	80-100 ns	180-200 ns	280-300 ns	100-300 ns	1-300 ns
WT-1	-25.8±2.9	-28.2±2.9	-28.6±3.4	-27.2±4.1	-26.7±3.7
WT-2	-30.4±4.1	-30.1±3.4	-27.8±3.5	-28.3±4.2	-29.0±4.1
Asn213Ala-1	-29.7±2.8	-27.6±2.8	-27.5±3.6	-28.2±3.7	-28.2±3.3
Asn213Ala-2	-26.5±3.5	--28.0±3.1	-28.3±3.2	-27.1±3.5	-27.2±3.4
Gly54Ala-1	-27.0±3.2	-26.9±3.2	-28.9±3.3	-27.5±3.0	-27.7±3.0
Gly54Ala-2	-28.5±3.2	-25.9±2.9	-26.7±3.1	-26.4±3.1	-26.9±3.3

Supplementary figures

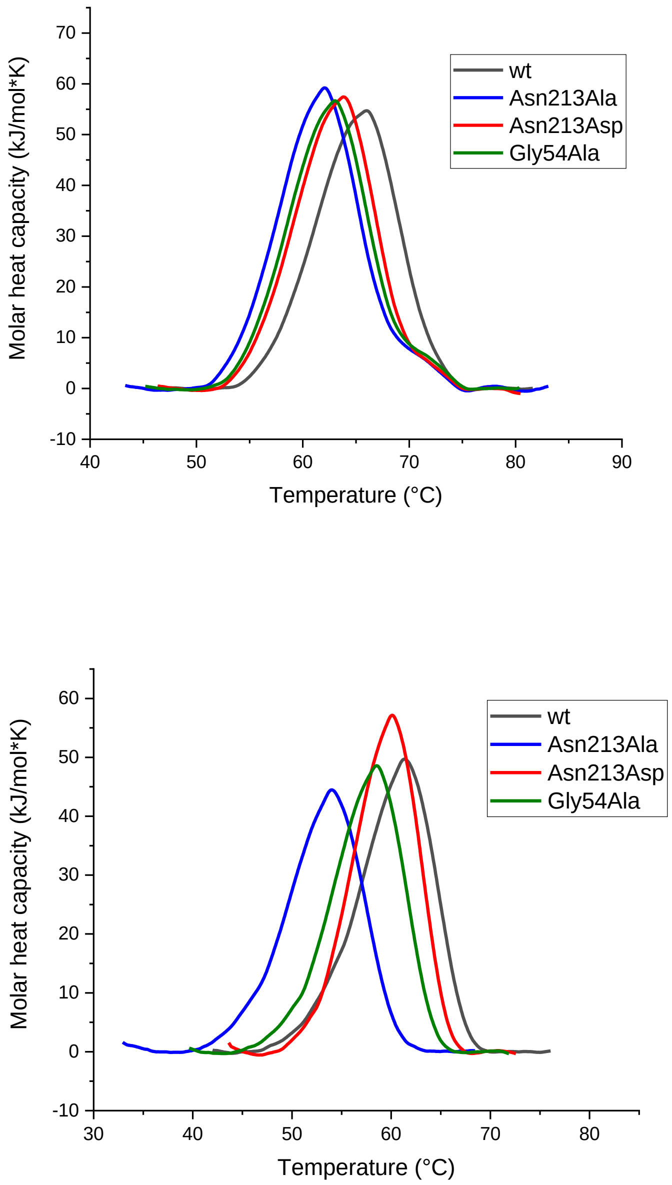

Figure S1. DSC thermograms of the wild-type SrL and its active mutated variants in buffer (10 mM phosphate, 200 mM NaCl, pH 8) (upper panel) and buffer/DMSO mixture (10 mM phosphate, 200 mM NaCl, pH 8, 20% DMSO) (lower panel).

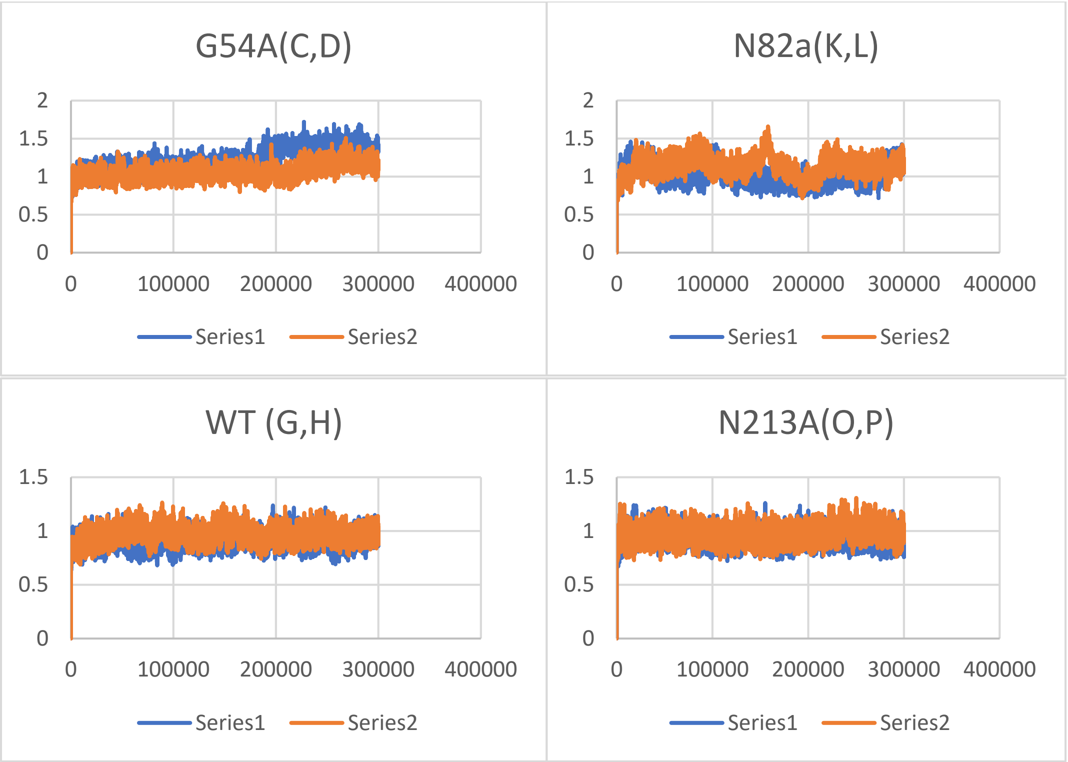

Figure S2. RMSD (Å) of protein backbone atoms during 300 ns of MD simulations of ligand-free SrL variants in water solution.

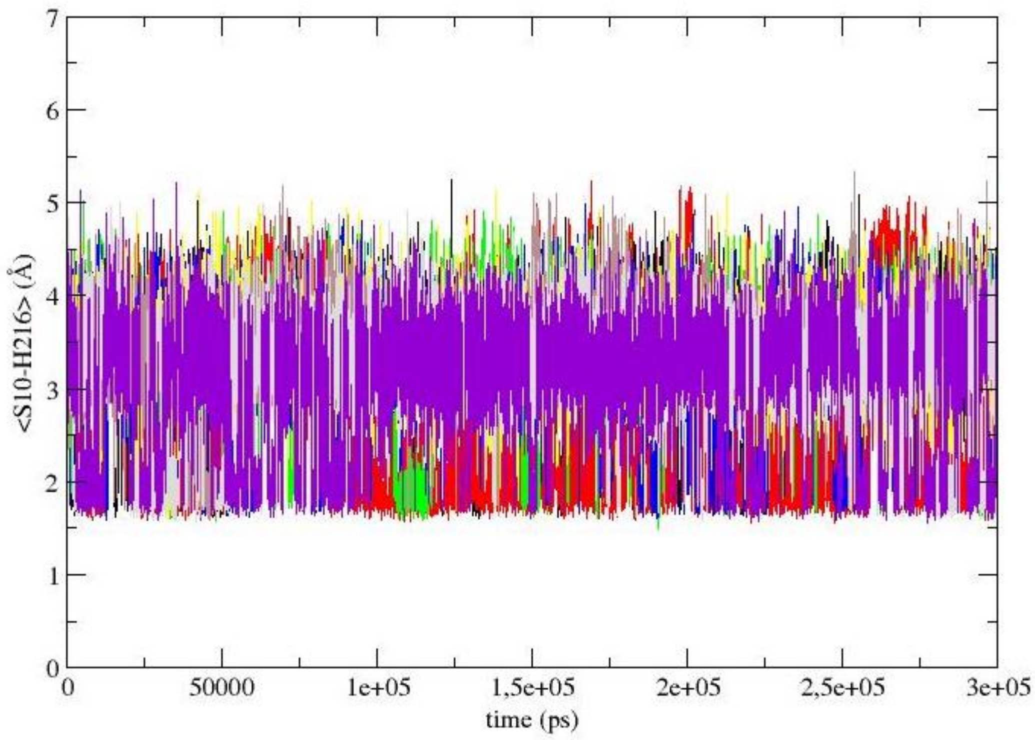

Figure S3. Distance between Ser10 and His216 during MD of ligand free SrL variants: WT (black and red), Asn213Ala (blue and green), Gly54Ala (yellow and brown), Asn82Ala (grey and violet).

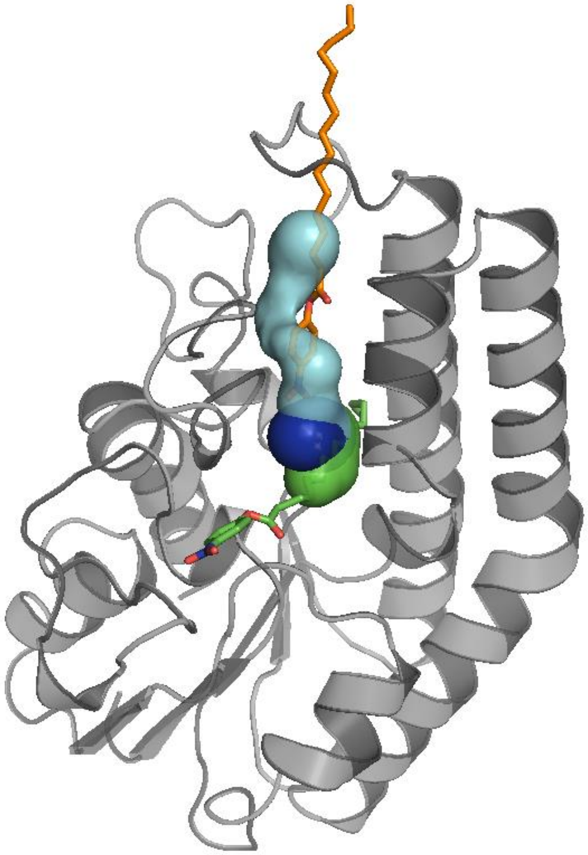

Figure S4. Tunnels determined by Caver (1, 2 and 3) coloured green, blue and cyan, respectively. Two initial structures used in ASMD simulations: with *p*NPP (green) accommodated in tunnel 1 and with *p*NPP (orange) accommodated on tunnel 3.

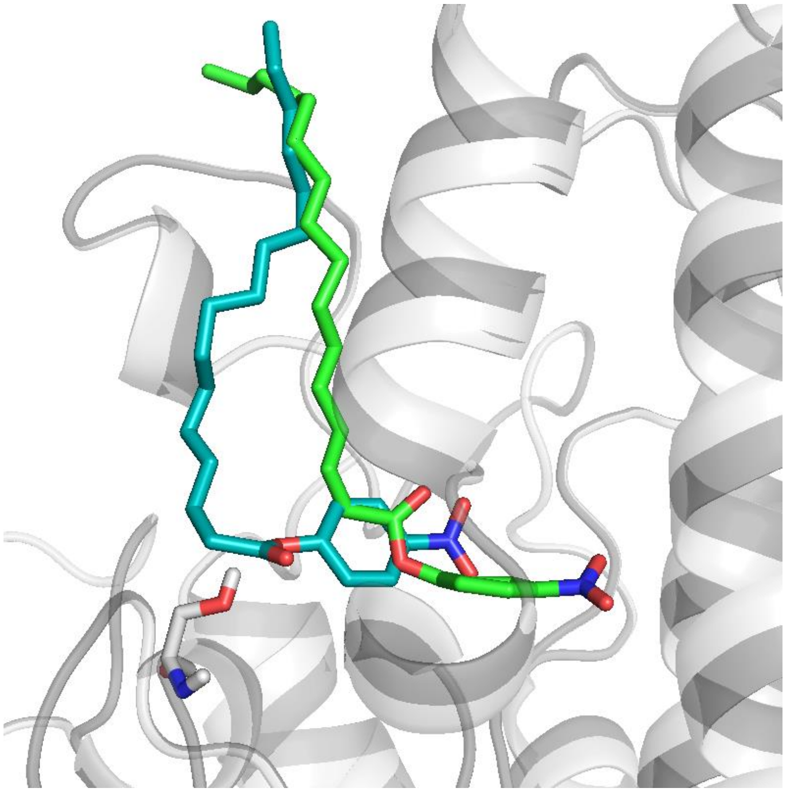

Figure S5. Orientation of the ligand in the SrL-*p*NPP complex obtained using ASMD simulation (cyan) for the *p*NPP alkyl chain accommodated in tunnel 3, and after additional 100 ns of unconstrained MD simulation of the solvated complex at room temperature (green). Position of Ser10 in the later (light grey) is shown in stick representation.

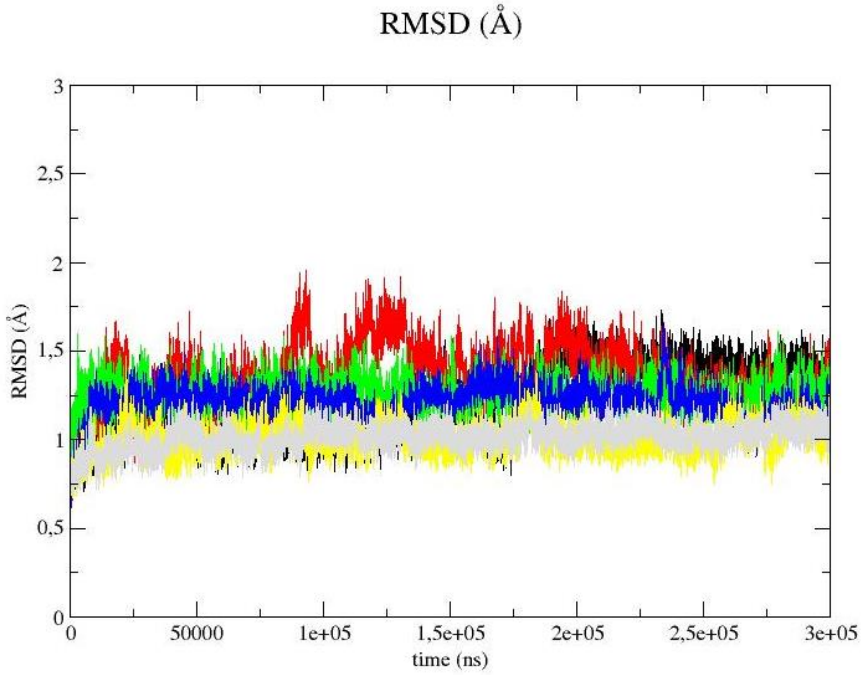

Figure S6. RMSD (Å) during 300 of MD simulations of the WT SrL (black and red lines) and its mutants Asn213Ala (blue and green lines) and Gly54Ala (grey and yellow lines). Simulations were performed at 300 K using OPC water molecules. RMSD is calculated for the protein heavy backbone atoms according to the initial structure.

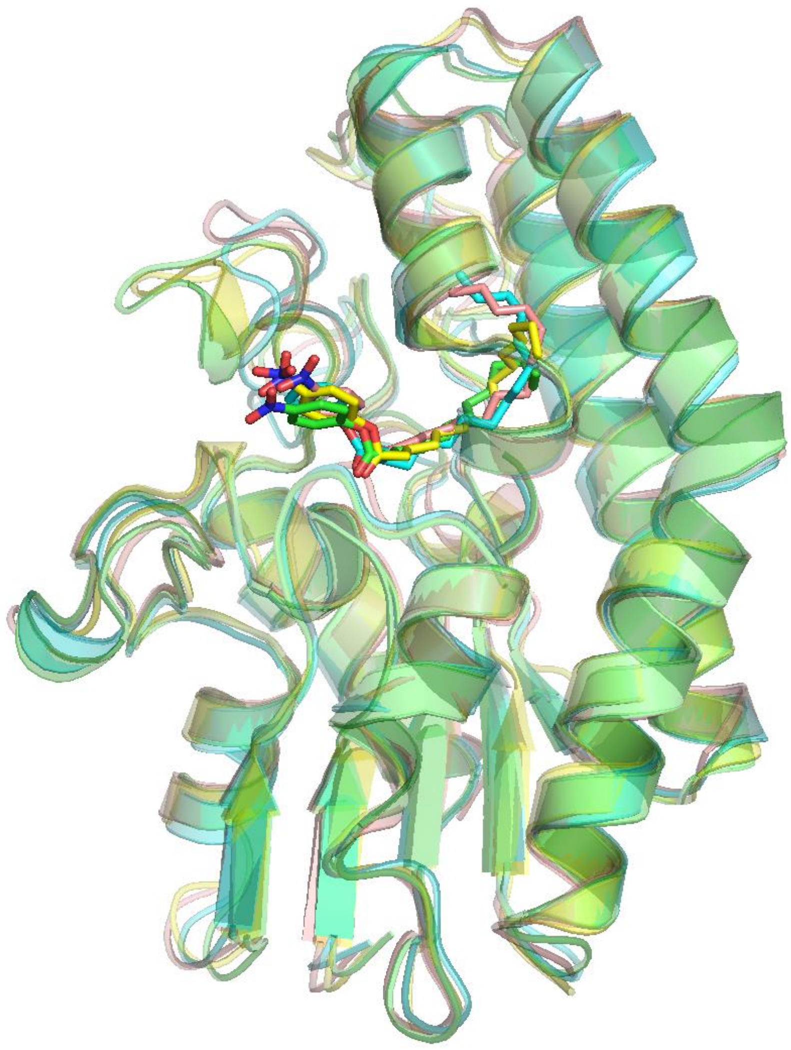

Figure S7. Overlay of the initial WT-*p*NPP structure (coloured green) and the structures of WT-*p*NPP (yellow), Gly54Ala-*p*NPP (orchard) and Asn213Ala-*p*NPP (cyan) obtained after 300 ns of MD simulations at 300K using OPC water molecules.

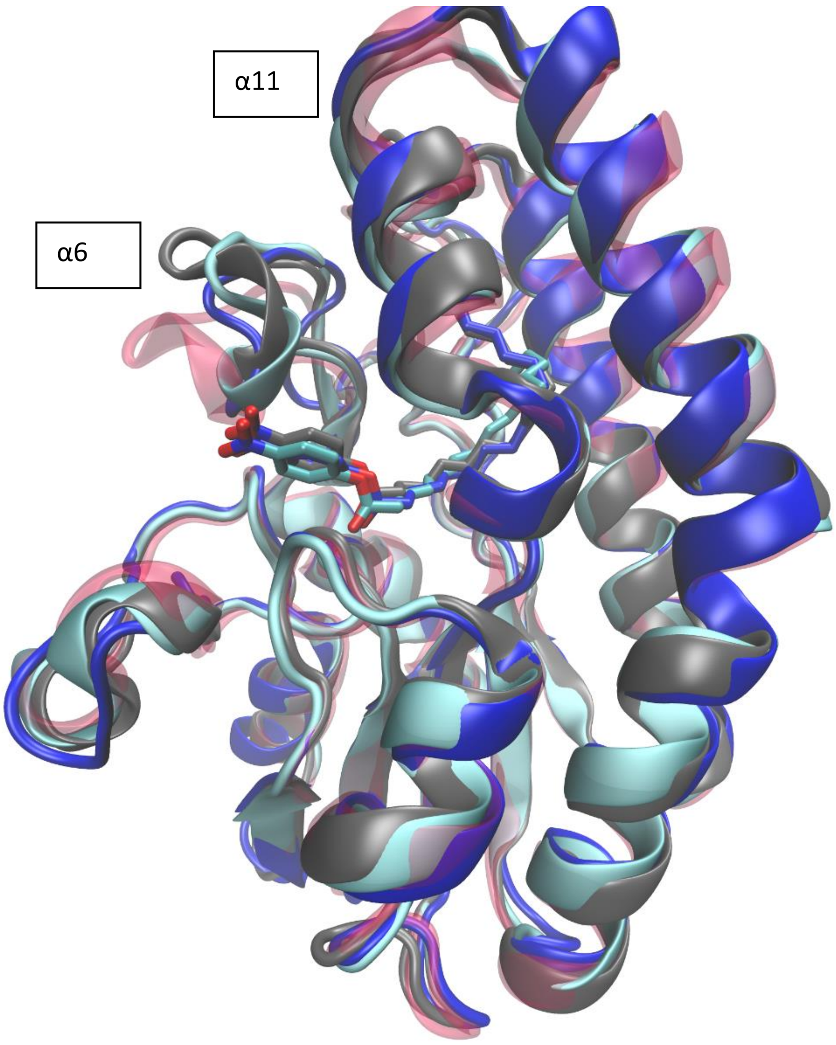

Figure S8. Overlay of the crystal structure of SrL (PDB_id 5mal) (coloured red) and the structures of two SrL variants (WT- coloured cyan and Asn213Ala mutant coloured blue) in the complex with *p*NPP obtained after 300 ns of MD simulations at 300K.

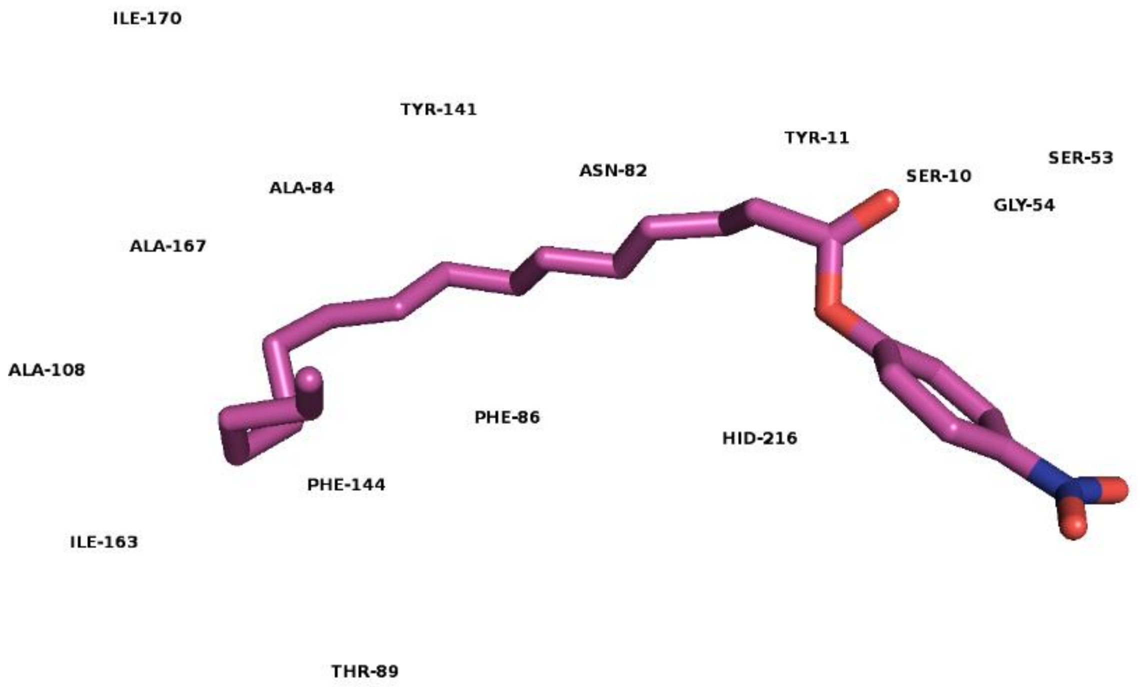

Figure S9. Amino acid residues that stabilize *p*NPP during simulations MD simulations starting from the complex obtained by ASMD simulation with the substrate initially accommodated in tunnel 1.

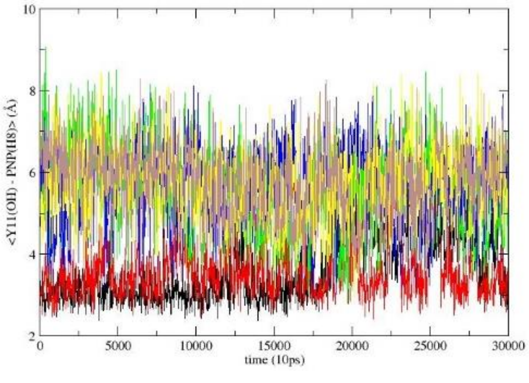
<Y11(OH) - PNP(H8)> (Å)
time (10ps)

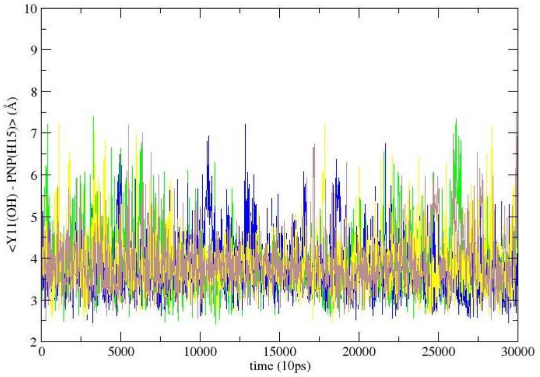
<Y11(OH) - PNP(H15)> (Å)
time (10ps)

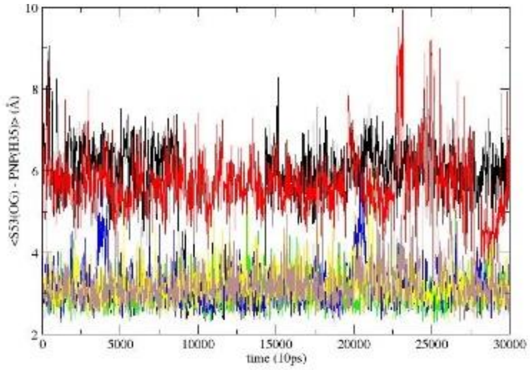
<S53(OG) - PNP(H35)> (Å)
time (10ps)

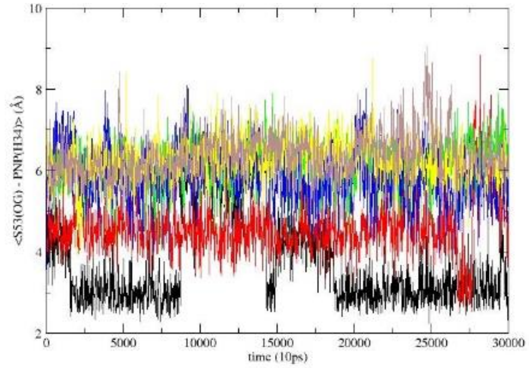
<S53(OG) - PNP(H34)> (Å)
time (10ps)

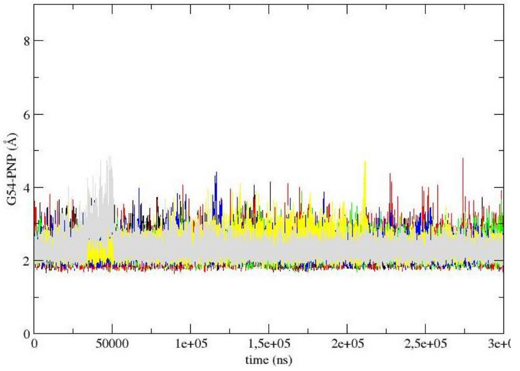
G54-PNP (Å)
time (ns)

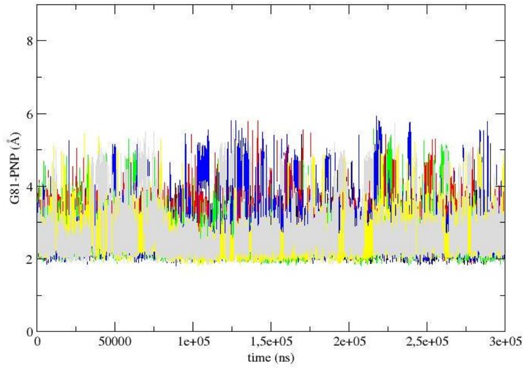
G81-PNP (Å)
time (ns)

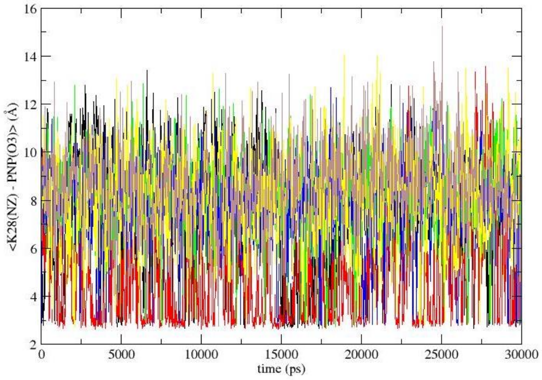
<K28(NZ) - PNP(O3)> (Å)
time (ps)

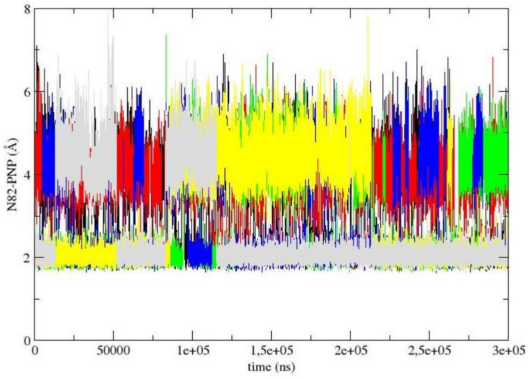
N82-PNP (Å)
time (ns)

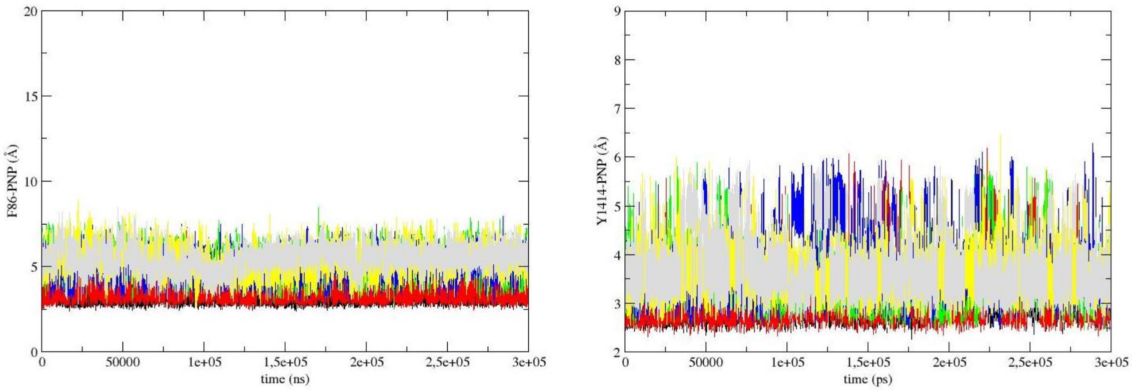

Figure S10. Distances between *p*NPP and some nearby amino acid residues during 300 ns of MD simulations at 300 K using OPC water molecules. The distances for runs 1 and 2 are shown as lines: for WT-*p*NPP (black and red), for Asn213Ala-*p*NPP (blue and green), for Gly54Ala-*p*NPP (yellow and grey).

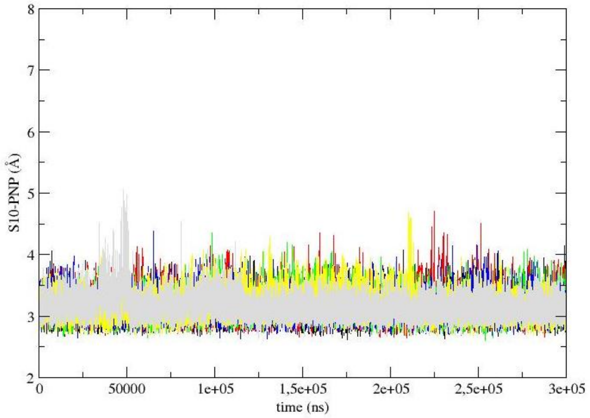

Figure S11. Distance between *p*NPP and Ser10 during 300 ns of MD simulations at 300 K using OPC water molecules. The distances for runs 1 and 2 are shown as lines: for WT-*p*NPP (black and red), for Asn213Ala-*p*NPP (blue and green), for Gly54Ala-*p*NPP (yellow and grey).

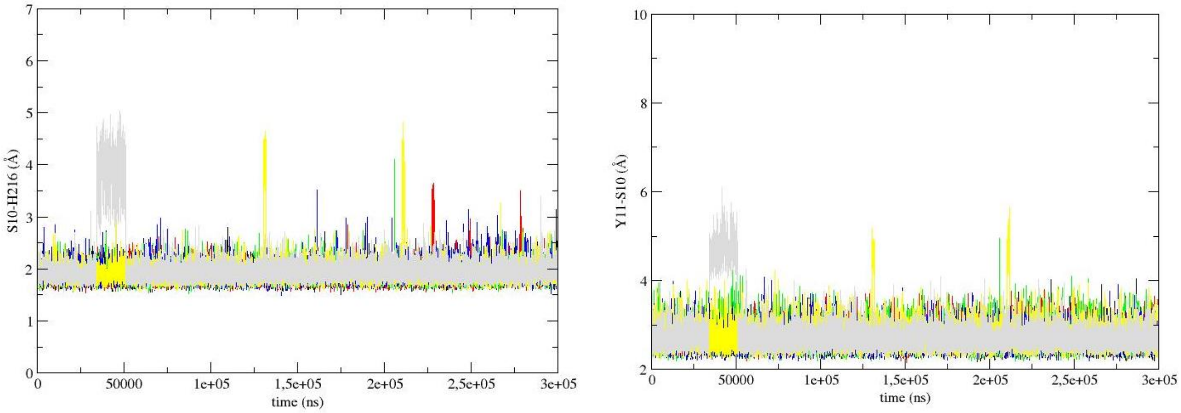

Figure S12. Distances between, Ser10 and His216 (left) and Ser10 and Tyr11 (right) during 300 ns of MD simulations at 300 K using OPC water molecules. Distances for runs 1 and 2 are shown as lines: for WT-*p*NPP (black and red), for Asn213Ala-*p*NPP (blue and green), for Gly54Ala-*p*NPP (yellow and grey).

Supplementary methods

Cloning

Wild type *srl* gene was amplified by PCR using forward primer srlEco, reverse primer srlHind (**Table S7**) and previously constructed pDJ5 as a template [10], **Table S8**. PCR mixture for the amplification of the *srl* gene contained: 0.8 mM primers, 200 mM dNTPs, 4 mM $MgSO_4$, 10% DMSO, 0.025 U of Pfu DNA Polymerase (Fermentas, USA) and 1 ng of DNA template per 25 µL of reaction mixture. Cycling conditions were: denaturation at 95°C for 3 min; following 28 cycles of denaturation at 95°C for 50 s, annealing at 69°C for 45 s, extension at 72°C for 1 min, and final extension at 72°C for 5 min. To enable subsequent gene cloning and protein purification steps, we have introduced *EcoRI* restriction site and ribosomal binding site upstream of the *srl* gene using the forward primer srlEco. Via reverse primer srlHind we have introduced six histidine GTG codons upstream of stop codon and *HindIII* restriction site downstream of stop codon (**Table S7**). The amplified 840 bp *srl* fragment was ligated into pGEM-T vector using pGEM®-T Vector System kit, following manufacturer's instructions (Promega, Madison, USA). Ligation mixture was used to transform electrocompetent *E. coli* XL1 cells. Plasmids were purified from the liquid cultures of the transformants with QIAprep Spin Miniprep Kit (QIAGEN, Germany) and the expected sequence of *srl* gene was confirmed by Sanger sequencing using T7f and pUCM13 / reverse primers (**Table S7**). The correct sequence of all lipase gene variants was confirmed by sequencing (Macrogen). Next, SrLpGEM plasmid were used for direct *EcoRI*/*HindIII* cloning of WT *srl* gene into pANT849pWB19N (as described previously by [38] obtaining the WT SrLpANT construct. Alternatively, WT SrLpGEM construct was used as a template to generate series of mutant *srl* genes.

Site-directed mutagenesis

Site-directed mutagenesis protocol used to produce six mutant variants of *srl* gene was based on protocols described by Xiao et al. [37] and QuickChange® XL Site-Directed Mutagenesis Kit Manual (Agilent Technologies, 2015) with some modifications. Four primers were used to produce each selected mutation: two flanking primers (T7f and pUCM13 / reverse) and two mutagenic primers specific for each mutation (**Table S7**). Both forward and reverse mutagenic primer annealed to the same region of the *srl* gene and contained the desired substitution of 1 or 2 nucleotides compared to the WT gene, positioned approximately in the middle of the primer

sequence. This enabled the change of one codon in the *srl* gene, i.e. one amino acid change in the corresponding SrL protein. Three PCR reactions were performed per mutation. PCR1a and PCR1b were performed to generate two fragments of *srl* gene ending in the desired mutation. PCR mixture (25 µL) contained 1 µM flanking primer, 0.2 µM mutagenic primer, 0.05 U of TaKaRa LA Taq Polymerase, 1X LA PCR Buffer GC I (Clontech Laboratories, Japan), 2.5 mM $MgCl_2$, 400 µM dNTP and 0.04 ng/µL of WT SrLpGEM as a template. Cycling parameters were: initial denaturation 94 °C / 1 min, followed by 28 cycles of denaturation at 98 °C / 10 sec, annealing at 50 °C / for 1 min and extension at 72 °C / 1 min, with final extension at 72 °C / 5 min. Next, PCR2 was performed with 10 to 50 ng of PCR1a and PCR1b amplicons in a 1:1 molar ratio as a template and two flanking primers. In this way, PCR1a and PCR1b fragments were combined in a single fragment which was subsequently ligated into pGEM-T vector to obtain a series of mutant SrLpGEM constructs. The expected sequence of mutant *srl* gene variants was confirmed by sequencing (Macrogen).

Table S6. Bacterial strains and culture media.

**Bacterial strains**	**Genotype**	**Purpose**
Escherichia coli XL1-Blue MRF '	*endA1 gyrA96(nal^R) thi-1 recA1 relA1 lac glnV44 e14- Δ(mcrCB-hsdSMR-mrr)171 recB recJ sbcC umuC::Tn5 uvrC F'[::Tn10 proAB$^+$ lacI^q Δ(lacZ)M15 Amy Cm^R]*	Cloning
Escherichia coli GM119	*dam-3 dcm-6 metBI gaIK2 gaIT22 lacYI tsx-78 thi-1 mtlA2*	Source of non-methylated DNA for *S. lividans* transformation
Streptomyces lividans TK23	SpcR	Heterologous production of wild type (WT) and mutant lipase variants
**Media**	**Composition/1 L**	**Source**
liquid GR_2d	7 g $CaCO_3$, 40 g dextrin, 8 g corn steep solids, 2 g $(NH_4)_2SO_4$, 280 µL lactic acid, 10 mL 1 M Tris-HCl, pH 8.0	[35]
liquid CRM	10 g glucose, 103 g sucrose, 10.12 g $MgCl_2 \cdot 6H_2O$, 15 g tryptic soy broth, 5 g yeast extract, distilled water to 1 L	[34]
solid MS	20 g mannitol, 20 g soya flour, 20 g agar, tap water to 1 L	[36]

solid R5	103 g sucrose, 0.25 g K_2SO_4, 10.12 g $MgCl_2 \cdot 6H_2O$, 10 g glucose, 0.1 g casamino acids, 2 mL trace element solution, 5 g yeast extract, 5.73 g TES buffer, 22 g Bacto agar, distilled water to 1 L; after autoclave sterilization add: 10 mL KH_2PO_4 (0,5%), 4 mL $CaCl_2 \cdot 2H_2O$ (5M), 15 mL L-proline (20%), 7 mL NaOH (1M)	[36]

Table S7. Oligonucleotide primers designed and used within this study. Restriction sites are underlined; RBS, start and stop codons are bolded; introduced mutations in mutagenic primers are bold and underlined.

Primer name	Sequence	Bp
srlEco	5' CGGAATTC**GGAGG**TTCC**ATG**AGACTGTCCCGACGCGCGGCCACGGCGT 3'	48
srlHind	5' ATATAAGCTT**TCA**(GTG)$_6$GGTGGCGGAGTTCAGGACGGG 3'	52
T7f	5' TAATACGACTCACTATAGGG 3'	20
pUCM13 / reverse	5' CAGGAAACAGCTATGAC 3'	17
Ser10AlaF	5' GTGGCCCTCGGCGAC**G**CCTACTCCTCGGGGGTC 3'	33
Ser10AlaR	5' GACCCCCGAGGAGTAGG**C**GTCGCCGAGGGCCAC 3'	33
Gly54AlaF	5' CACCGCCTGTTCGG**C**CGCCCGCACAGGAG 3'	29
Gly54AlaR	5' CTCCTGTGCGGGCG**G**CCGAACAGGCGGTG 3'	29
Asn82AlaF	5' CATTACCATCGGCGGC**GC**CGACGCGGGCTTCGCC 3'	34
Asn82AlaR	5' GGCGAAGCCCGCGTCG**GC**GCCGCCGATGGTAATG 3'	34
Asn213AlaF	5' CCCTTCCCGTGGAG**GC**CTCCTACCACCCCACG 3'	32
Asn213AlaR	5' CGTGGGGTGGTAGGAG**GC**CTCCACGGGAAGGG 3'	32
Asn213AspF	5' CCCTTCCCGTGGAG**G**ACTCCTACCACCCCACG 3'	32
Asn213AspR	5' CGTGGGGTGGTAGGAGT**C**CTCCACGGGAAGGG 3'	32
His216AlaF	5' GTGGAGAACTCCTAC**GC**CCCCACGGCCAACGGACAG 3'	36
His216AlaF	5' CTGTCCGTTGGCCGTGGGG**GC**GTAGGAGTTCTCCAC 3'	36

Table S8. Plasmids.

Plasmid name	Description/Purpose	Source
pDJ5	Contains the WT *srl* gene within a 4-kb BamHI fragment in pMTL23	[10]
pGEM-T	*E. coli* plasmid used for TA-cloning and sequencing of mutated *srl* gene variants before re-cloning into pANT849	pGEM-T Easy vector system kit (Promega)
pANT849pWB19N	Bifunctional *E. coli*/*Streptomyces* vector for cloning of WT *srl* gene gene and its mutant variants	[38]
SrLpGEM	pGEM-T containing *srl* gene (WT and series of mutants)	This study
SrLpANT	pANT849 containing *srl* gene and its mutated variants used for heterologous overexpression lipases	This study

References: All references listed in the Supplementary are listed in the main manuscript.
